# Robust Sparse Bayesian Two-Dimensional Direction-of-Arrival Estimation with Gain-Phase Errors

**DOI:** 10.3390/s23239422

**Published:** 2023-11-26

**Authors:** Xu Jin, Xuhu Wang, Yujun Hou, Siyuan Hao, Xinjie Wang, Zhenhua Xu, Qunfei Zhang

**Affiliations:** 1 School of Information and Control Engineering, Qingdao University of Technology, Qingdao 266520, China; jinxu@qut.edu.cn (X.J.); houyujun@qut.edu.cn (Y.H.); wangxinjie@qut.edu.cn (X.W.); 2 Institute of Oceanology, Chinese Academy of Sciences, Qingdao 266071, China; xuzhenhua@qdio.ac.cn; 3 School of Software Engineering, Beijing Jiaotong University, Beijing 100044, China; syhao@bjtu.edu.cn; 4 School of Marine Science and Technology, Northwest Polytechnical University, Xi’an 710129, China; zhangqf@nwpu.edu.cn

**Keywords:** two-dimensional direction-of-arrival (2D DOA), gain-phase errors, sparse signal reconstruction, sparse Bayesian learning, L-shaped array

## Abstract

To reduce the influence of gain-phase errors and improve the performance of direction-of-arrival (DOA) estimation, a robust sparse Bayesian two-dimensional (2D) DOA estimation method with gain-phase errors is proposed for L-shaped sensor arrays. The proposed method introduces an auxiliary angle to transform the 2D DOA estimation problem into two 1D angle estimation problems. A sparse representation model with gain-phase errors is constructed using the diagonal element vector of the cross-correlation covariance matrix of two submatrices of the L-shaped sensor array. The expectation maximization algorithm derives unknown parameter expression, which is used for iterative operations to obtain off-grid and signal precision. Using these parameters, a new spatial spectral function is constructed to estimate the auxiliary angle. The obtained auxiliary angle is substituted into a sparse representation model with gain and phase errors, and then the sparse Bayesian learning method is used to estimate the elevation angle of the incident signal. Finally, according to the relationship of the three angles, the azimuth angle can be estimated. The simulation results show that the proposed method can effectively realize the automatic matching of the azimuth and elevation angles of the incident signal, and improves the accuracy of DOA estimation and angular resolution.

## 1. Introduction

The estimation of the direction-of-arrival of sensor arrays is an important research direction in the fields of radar, sonar and mobile communications, which has attracted the interest of many scholars [1,2,3]. At present, the research on uniform linear arrays (ULAs) is relatively mature, and many high-resolution DOA estimation methods have been proposed to improve the accuracy and resolution, such as the subspace method [4,5], maximum likelihood methods [6,7] and compressed sensing methods. Compared with the first two types of methods, the DOA estimation method based on compressed sensing can effectively estimate the incident angle in the case of a few snapshots and low signal-to-noise ratio (SNR). The reconstruction methods of compressed sensing technology are mainly divided into three categories: convex optimization methods [8,9], greedy iteration methods [10,11] and sparse Bayesian learning (SBL) methods.

Among these methods, by exploring the spatial sparsity of incident signals, sparse Bayesian learning methods have better adaptive performance than the other traditional methods under the conditions of a limited number of snapshots, low SNR and spatially adjacent signals. The SBL method [12], compared with convex optimization methods, not only has the same global convergence, but also has relatively higher computational efficiency. Although the SBL estimation method improves the resolution, its estimation performance is limited by the degree of spatial dispersion. Increasing the estimation accuracy by adding a preset grid will bring a huge computational burden; however, the estimation accuracy will be reduced without increasing the preset grid, that is, there is a mesh mismatch problem. Therefore, Yang et al. [13] considered introducing an off-grid model and proposed an off-grid Sparse Bayesian Inference (OGSBI) method, which further reduced the influence of grid mismatch and improved the DOA estimation accuracy. Dai et al. [14] proposed a root-off grid sparse Bayesian learning (RootSBL) DOA estimation method, which improves the operation efficiency by ensuring high direction estimation accuracy. However, because the off-grid models in the above two methods use first-order Taylor expansion, their estimation performance under a coarse grid is not satisfactory. To solve this problem, the authors of [15] introduced second-order Taylor approximation into off-grid DOA estimation. Although the higher-order Taylor approximation reduces the approximate error and improves the accuracy of DOA estimation, it also increases the computational complexity. In order to reduce the amount of computation, Refs. [16,17] introduced different forms of real-value transformation, using compressed sensing technology to transform the data in the complex number field into the real number field and reduce the operation time of each iteration. Then, for different application backgrounds such as color noise and broadband signals, many researchers used the DOA estimation methods based on variational sparse Bayes [18,19,20], and the DOA estimation performances of such methods were further improved using SBL. Nevertheless, the above methods based on sparse Bayesian learning are all based on ULAs, which can only provide one-dimensional angle information. In practical applications, it is more useful to obtain the 2D direction of the source, that is, the azimuth angle and the elevation angle. In recent years, the research on 2D DOA estimation has gradually increased, and scholars have proposed many array geometries for 2D estimation, such as circular arrays, rectangular arrays and cross arrays. Compared with these 2D arrays, L-shaped arrays are simpler in structure, easier to implement, and have better estimation performance, which has attracted much attention [21].

Although the above types of estimation methods have good DOA estimation performances, they depend on the ideal array manifold, while in practical application scenarios, array manifolds are often affected by unknown gain-phase errors. Without array manifold calibration, the performance of DOA estimation may be greatly degraded [22]. Therefore, the study of DOA estimation methods with array gain-phase errors has theoretical significance and practical value. Of the existing methods to solve the gain-phase error problem, there are two main categories: pre-calibration and self-calibration methods. Pre-calibration methods generally require placing an additional pre-calibration source, and the pre-calibration source needs to have accurate bearing information. The placement of the pre-calibration source is used to estimate the gain phase error and correct the DOA [23,24]. However, in practical engineering applications, it is not easy to ensure the existence of calibration sources, so it is not suitable for practical engineering applications. However, self-calibration methods can directly estimate the gain-phase error during array operation without placing the pre-calibration source [25,26,27]. Although these methods usually adopt iterative methods and require a large amount of computation, compared with pre-calibration methods, it is more likely to be implemented in actual engineering.

In order to solve the problem of low accuracy and poor angular resolution caused by unknown gain-phase errors, a robust sparse Bayesian two-dimensional DOA estimation method with gain-phase errors is proposed by combining L-shaped matrices. The main contributions of this method are as follows:

- A new auxiliary angle is introduced to transform the 2D DOA estimation problem into two 1D DOA estimation problems to reduce the computational complexity.
- For the sparse reconstruction problem with gain-phase errors, the expected maximum algorithm is used to derive the estimation expressions of all unknown parameters to obtain new spatial spectral functions and the angle information to overcome the gain-phase errors.
- By solving two sparse reconstruction problems with gain-phase errors, the auxiliary angle and its corresponding elevation angle are estimated and then the azimuth angle estimation and automatic angle matching are completed according to the relationship between the three angles.

Notation: The superscripts ${\left(\cdot \right)}^{T}$, ${\left(\cdot \right)}^{H}$, and ${\left(\cdot \right)}^{*}$ denote the transpose, the conjugate transpose, and the conjugate, respectively. ${\left(\cdot \right)}^{-1}$ denotes matrix inversion. diag(⋅) returns a diagonal matrix whose main diagonal is given in the bracket and blkdiag(⋅) represents the block diagonal matrix. ℜ{⋅} denotes the real part of a complex value. Tr{⋅} represents the trace operation of a matrix, and ${\left\|\cdot \right\|}_{2}$ denotes the $l_{2}$ norm. The mathematical symbols employed throughout the manuscript are listed in Table 1.

## 2. Signal Model

**Figure 1:**
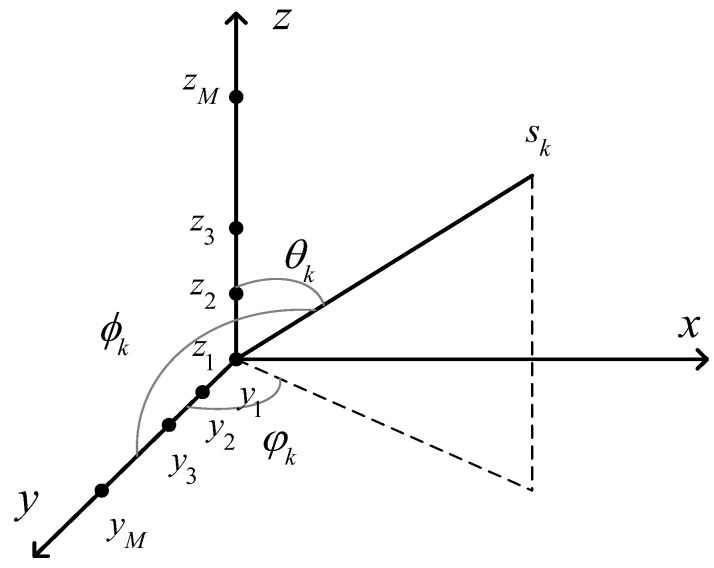
Uniform L-shaped array.

In practical applications, 2D DOA estimation is closer to the directional characteristics of real electromagnetic propagation signals, and is usually based on uniform rectangular arrays (URAs) and uniform circular arrays (UCAs). However, compared with URAs and UCAs, L-shaped arrays covers a larger area and have a wider application range, so this study used uniform L-shaped arrays, as shown in Figure 1. The subarray on the y axis consists of a uniform linear array with M elements; the element spacing of the ULA is d=λ/2, where λ is the wavelength of the signal; and the subarray on the z-axis has the same structure as the subarray on the *y*-axis.

Suppose that K far-field narrowband sources $s_{k}\left(t\right)$ are incident on a ULA equipped with 2M−1 array elements. The incoming wave direction of the incident signal is $\left(\theta_{k},\phi_{k}\right)$,k=1,2,⋯,K, $\theta_{k}$ is the elevation angle, $\phi_{k}$ is the azimuth angle and the number of snapshots is T; then, the model of the received signal of the subarray on the *z*-axis and *y*-axis can be expressed as

(1) $$z\left(t\right)=A\left(\theta \right)s\left(t\right)+n_{z}\left(t\right),$$

(2) $$y\left(t\right)=A\left(\phi \right)s\left(t\right)+n_{y}\left(t\right),$$

where $z\left(t\right)={\left[z_{1}\left(t\right),z_{2}\left(t\right),\cdots ,z_{M}\left(t\right)\right]}^{T}$ and $y\left(t\right)={\left[y_{1}\left(t\right),y_{2}\left(t\right),\cdots ,y_{M}\left(t\right)\right]}^{T}$ represents the array receiving signals on the z and y axes, respectively; $s\left(t\right)={\left[s_{1}\left(t\right),s_{2}\left(t\right),\cdots ,s_{K}\left(t\right)\right]}^{T}$ represents the incident signal, where “T” denotes the transpose operator; both $n_{z}\left(t\right)$ and $n_{x}\left(t\right)$ stand for the complex additive Gaussian white noise vector with zero means that are independent from the incident signal. $A\left(\theta \right)=[a\left(\theta_{1}\right),a\left(\theta_{2}\right),\cdots ,a\left(\theta_{K}\right)]$ and $A\left(\phi \right)=[a\left(\phi_{1}\right),a\left(\phi_{2}\right),\cdots ,a\left(\phi_{K}\right)]$ indicate the ideal array manifold matrix, where $a\left(\theta_{k}\right)$ and $a\left(\phi_{k}\right)$ satisfy the following:

(3) $$a\left(\theta_{k}\right)={\left[1,e^{\frac{-j2\pi d\cos\theta_{k}}{\lambda }},\cdots ,e^{\frac{-j2\pi \left(M-1\right)d\cos\theta_{k}}{\lambda }}\right]}^{T},$$

(4) $$a\left(\phi_{k}\right)={\left[1,e^{\frac{-j2\pi d\cos\phi_{k}}{\lambda }},\cdots ,e^{\frac{-j2\pi \left(M-1\right)d\cos\phi_{k}}{\lambda }}\right]}^{T}.$$

In the case of multiple snapshots, the array signal models can be expressed in matrix form as follows:

(5) $$Z=A_{z}S+N_{z},$$

(6) $$Y=A_{y}S+N_{y}.$$

At present, most signal processing methods are based on the ideal condition of the array manifold, but gain-phase errors may exist in practical applications. Therefore, considering the condition of gain-phase errors, the array signal models are expressed as

(7) $$Z=\Gamma_{z}A_{z}S+N_{z},$$

(8) $$Y=\Gamma_{y}A_{y}S+N_{y},$$

where $\Gamma_{z}=\mathrm{diag}\left(\rho_{z1}e^{j\varphi_{z1}},\cdots ,\rho_{zm}e^{j\varphi_{zm}},\cdots ,\rho_{zM}e^{j\varphi_{zM}}\right)$ and $\Gamma_{y}=\mathrm{diag}\left(\rho_{y1}e^{j\varphi_{y1}},\cdots ,\rho_{ym}e^{j\varphi_{ym}},\cdots ,\rho_{yM}e^{j\varphi_{yM}}\right)$ are the two matrices of the gain-phase error coefficients between subarray elements, and $\rho_{m}$ and $\varphi_{m}$ denote the gain error and the phase error of the mth array, respectively.

From the above statement, it is clear that the elevation angles and azimuth angles are estimated completely separately. However, if the elevation angles and azimuth angles are estimated separately, and then additional angle matching is performed, the overall performance of the method will be poor at low SNRs and with small snapshots. Therefore, we consider an auxiliary angle $\eta_{k}$ which satisfies $\cos\eta_{k}=\cos\phi_{k}-\cos\theta_{k}$, and then the received data can be expressed as

(9) $$X=\left[\begin{array}{c} Z\\ Y \end{array}\right]=\left[\begin{array}{c} \Gamma_{z}A_{z}\\ \Gamma_{y}A_{y} \end{array}\right]S+\left[\begin{array}{c} N_{z}\\ N_{y} \end{array}\right]=\Gamma A\left(\theta ,\phi \right)S+N,$$

where $\Gamma =\mathrm{blkdiag}\left(\Gamma_{z},\Gamma_{x}\right)$, $A\left(\theta ,\phi \right)=\left[a\left(\theta_{1},\phi_{1}\right),\cdots ,a\left(\theta_{K},\phi_{K}\right)\right]$ and $a\left(\theta_{k},\phi_{k}\right)={\left[a^{T}\left(\theta_{k}\right),a^{T}\left(\phi_{k}\right)\right]}^{T}$; based on the relationship between the three angles, the new direction vector can be expressed as $a\left(\theta_{k},\phi_{k}\right)=\Theta \left(\eta_{k}\right)a\left(\theta_{k}\right)$, $\Theta \left(\eta_{k}\right)={\left[I_{M},\mathrm{diag}\left(e{\left(\eta_{k}\right)}^{*}\right)\right]}^{T}$ and $e\left(\eta_{k}\right)=\left[1,e^{-j2\pi d\cos\eta_{k}},\cdots ,e^{-j2\pi d\left(M-1\right)\cos\eta_{k}}\right]$.

## 3. The Proposed Method

### 3.1. Off-Grid Model

Let $\bar{\theta }=\{{\bar{\theta }}_{1},{\bar{\theta }}_{2},\cdots ,{\bar{\theta }}_{N}\}$ be a fixed sampling grid in the DOA range $\left[-90^{{}^{\circ}},90^{{}^{\circ}}\right]$, where N denotes the grid number and typically satisfies N>>M>K. Assuming that the source is incident to the preset grid point without bias, that is $\theta_{k}\in \bar{\theta }$, the general array signal model with gain-phase errors can be expressed as

(10) $$X=\Gamma A\left(\bar{\theta }\right)\bar{S}+N,$$

where $A\left(\bar{\theta }\right)=[a\left({\bar{\theta }}_{1}\right),a\left({\bar{\theta }}_{2}\right),\cdots ,a\left({\bar{\theta }}_{N}\right)]$ is the over-complete dictionary set, and $\bar{S}$ is the row sparse signal matrix obtained after extension.

Since, in actual situations, the real incident signal position is not likely to be located exactly on the preset discrete spatial grid, we introduce an off-grid model. Let ${\bar{\theta }}_{k}$ represent the uniform grid with a grid interval $r={\bar{\theta }}_{2}-{\bar{\theta }}_{1}$ that we set up in advance. $\theta_{k}\notin \left\{{\bar{\theta }}_{1},{\bar{\theta }}_{2},\cdots ,{\bar{\theta }}_{N}\right\}$ denotes the real signal that does not belong to the set of preset discrete angles, and the first-order Taylor expansion of its steering vector can be approximated by

(11) $$\begin{array}{l} a\left(\theta_{k}\right)=a\left({\bar{\theta }}_{nk}\right)+b\left({\bar{\theta }}_{nk}\right)\left(\theta_{k}-{\bar{\theta }}_{nk}\right)\\ =a\left({\bar{\theta }}_{nk}\right)+b\left({\bar{\theta }}_{nk}\right)\beta_{nk} \end{array},$$

where ${\bar{\theta }}_{nk}$ represents the preset grid point closest to $\theta_{k}$, $\beta_{nk}=\theta_{k}-{\bar{\theta }}_{nk}$, and $b\left({\bar{\theta }}_{nk}\right)=a\prime \left({\bar{\theta }}_{nk}\right)$ represents the first derivative of $a\left({\bar{\theta }}_{nk}\right)$. Expressed in matrix form, $A\left(\bar{\theta }\right)=[a\left({\bar{\theta }}_{1}\right),a\left({\bar{\theta }}_{2}\right),\cdots ,a\left({\bar{\theta }}_{N}\right)]$, $B\left(\bar{\theta }\right)=[b\left({\bar{\theta }}_{1}\right),b\left({\bar{\theta }}_{2}\right),\cdots ,b\left({\bar{\theta }}_{N}\right)]$ and $\beta =[\beta_{1},\cdots ,\beta_{N}]$, $A\left(\bar{\theta }\right)$ in Equation (10) can be expressed as

(12) $$\Phi \left(\beta \right)=A\left(\bar{\theta }\right)+B\left(\bar{\theta }\right)\mathrm{diag}\left(\beta \right).$$

Therefore, Equation (10) can be further updated as

(13) $$X=\Gamma \Phi \left(\beta \right)\bar{S}+N=\Gamma \left[A\left(\bar{\theta }\right)+B\left(\bar{\theta }\right)\mathrm{diag}\left(\beta \right)\right]\bar{S}+N$$

**Lemma** **1.** *For any* N×1 *dimensional complex vector* a *and any* N×N *dimensional complex diagonal matrix* Γ, Γa=diag(a)γ *can be obtained, where* γ *is the element on the main diagonal of the complex diagonal matrix* Γ, i.e., $\gamma_{i}=\Gamma_{i,i}$.

According to Lemma 1, we obtain

(14) $$\Gamma A\left(\bar{\theta }\right)=\Gamma [a\left({\bar{\theta }}_{1}\right),a\left({\bar{\theta }}_{2}\right),\cdots ,a\left({\bar{\theta }}_{N}\right)]=A_{\Lambda }\left(I_{N}⊗\gamma \right),$$

(15) $$\Gamma B\left(\bar{\theta }\right)\mathrm{diag}\left(\beta \right)=\Gamma [b\left({\bar{\theta }}_{1}\right),b\left({\bar{\theta }}_{2}\right),\cdots ,b\left({\bar{\theta }}_{N}\right)]\mathrm{diag}(\beta )=B_{\Lambda }\left(I_{N}⊗\gamma \right)\mathrm{diag}\left(\beta \right),$$

where $A_{\Lambda }=\left[\mathrm{diag}\left(a\left({\bar{\theta }}_{1}\right)\right),\mathrm{diag}\left(a\left({\bar{\theta }}_{2}\right)\right),\cdots ,\mathrm{diag}\left(a\left({\bar{\theta }}_{N}\right)\right)\right]$, $I_{N}$ represents the N×N dimensional identity matrix, and ⊗ denotes the Kronecker product operation: $B_{\Lambda }=[B_{\Lambda 1},B_{\Lambda 2},\cdots ,B_{\Lambda N}]=\left[\mathrm{diag}\left(b\left({\bar{\theta }}_{1}\right)\right),\mathrm{diag}\left(b\left({\bar{\theta }}_{2}\right)\right),\cdots ,\mathrm{diag}\left(b\left({\bar{\theta }}_{N}\right)\right)\right]$, which can be obtained by summing Equations (14) and (15):

(16) $$\Gamma \left[A\left(\bar{\theta }\right)+B\left(\bar{\theta }\right)\mathrm{diag}\left(\beta \right)\right]\bar{S}=\left(A_{\Lambda }+B_{\Lambda }\left(\mathrm{diag}\left(\beta \right)⊗I_{M}\right)\right)\left(\bar{S}⊗\gamma \right).$$

By setting $\Psi \left(\beta \right)=\left(A_{\Lambda }+B_{\Lambda }\left(\mathrm{diag}\left(\beta \right)⊗I_{M}\right)\right)$, the sparse representation model of the array signal with gain-phase errors can be rewritten as

(17) $$X=\Psi \left(\beta \right)\left(\bar{S}⊗\gamma \right)+N.$$

To further simplify the above equation, let $\Omega \left(\beta ,\gamma \right)=\Psi \left(\beta \right)\left(\bar{S}⊗\gamma \right)$.

### 3.2. Hierarchical Sparse Bayesian Framework

First, it is assumed that the signal received by the array is independent between different snapshots, and the noise is circular symmetric complex Gaussian white noise, which follows a Gaussian distribution. Thus, we obtain

(18) $$p\left(N|\alpha_{0}\right)=\prod_{t=1}^{T}CN(n(t)|0,\alpha_{0}^{-1}I),$$

where $\alpha_{0}^{-1}≜\sigma_{n}^{2}$ is the variance of noise. The complex Gaussian distributed random variable with mean μ and covariance Σ obeys the probability density function of CN(μ,Σ) as

(19) $$CN\left(x|\mu ,\Sigma \right)=\frac{1}{\pi^{N}\left|\Sigma \right|}\exp\left\{-{\left(x-\mu \right)}^{H}\Sigma^{-1}\left(x-\mu \right)\right\}.$$

where “|⋅|”means taking the determinant.

In this paper, the variance of noise is unknown, so it should be assumed. Since the conjugate of the inverse of the variance of the Gaussian distribution is a Gamma distribution [28], a Gamma distribution is specified for the hyperparameter $\alpha_{0}$, which can be described as

(20) $$p\left(\alpha_{0}\right)=\Gamma \left(\alpha_{0};a,b\right)={\left[\Gamma \left(a\right)\right]}^{-1}b^{a}\alpha_{0}^{a-1}\exp\left\{-b\alpha_{0}\right\},$$

where a and b are the hyperparameters of $\alpha_{0}$, $\Gamma \left(a\right)=\int {}_{0}^{\infty }x^{a-1}e^{-x}dx$.

For received signals that are independent of each other, the sparse matrix $\bar{S}$ is assumed to follow a Gaussian distribution with a zero mean:

(21) $$p\left(\bar{S}|\alpha_{s}\right)=\prod_{t=1}^{T}CN\left({\bar{s}}_{t}|0,\Lambda \right),\begin{array}{cc} & \Lambda =\mathrm{diag}\left(\alpha_{s}\right) \end{array}.$$

Let the precision vector $\alpha_{s}={\left[\alpha_{s1},\alpha_{s2},\cdots ,\alpha_{sN}\right]}^{T}$ be a hyperparameter, which controls the estimation accuracy of the parameter $\bar{S}$ to be estimated. Then, $\alpha_{s}$ can be expressed by the Gamma prior with hyperparameters c and d:

(22) $$p(\alpha_{s})=\prod_{n=1}^{N}\Gamma \left(\alpha_{sn}|c,d\right).$$

For the case where the gain-phase error is unknown, and assuming that the gain phase error vector coefficients between arrays are independent of each other, the error vector distribution can also be expressed as a Gaussian distribution:

(23) $$p\left(\gamma |\alpha_{\gamma }\right)=\prod_{m=1}^{M}CN\left(\gamma_{m}|0,\Lambda_{\gamma }\right),\begin{array}{cc} & \Lambda_{\gamma }=\mathrm{diag}\left(\alpha_{\gamma }\right) \end{array}.$$

where $\alpha_{\gamma }={\left[\alpha_{\gamma 1},\alpha_{\gamma 2},\cdots ,\alpha_{\gamma M}\right]}^{T}$ represents the gain-phase error precision, and its distribution is described by Gamma distributions with hyperparameters e and f:

(24) $$p\left(\alpha_{\gamma };e,f\right)=\prod_{m=1}^{M}\Gamma \left(\alpha_{\gamma m};e,f\right).$$

Then, the off-grid vector is assumed; if it follows a uniform distribution, then it can be expressed as

(25) $$p\left(\beta \right)=U\left(\left[-\frac{1}{2}r,\frac{1}{2}r\right]\right),$$

where

(26) $$U\left(\left[a,b\right]\right)=\{\begin{array}{c} \frac{1}{b-a},\begin{array}{cc} & a\leq x\leq b \end{array}\\ 0,\begin{array}{cc} & otherwise \end{array} \end{array}.$$

### 3.3. Sparse Bayesian Inference

In this subsection, a self-correcting DOA estimation method for the unknown gain-phase error is proposed that uses sparse Bayesian inference under the assumption of the unknown parameter’s distribution in the previous section.

First, we give the following equation to maximize the posterior probability:

(27) $$\left\{\bar{S},\beta ,\gamma ,\alpha_{0},\alpha_{s},\alpha_{\gamma }\right\}=\underset{\left\{\bar{S},\beta ,\gamma ,\alpha_{0},\alpha_{s},\alpha_{\gamma }\right\}}{\arg\max}p\left(\bar{S},\beta ,\gamma ,\alpha_{0},\alpha_{s},\alpha_{\gamma }|X\right).$$

However, since this posterior probability problem is too complicated to be solved directly, the expectation maximization (EM) algorithm is considered here to obtain a solution.

(28) $$p\left(X,\bar{S},\beta ,\gamma ,\alpha_{0},\alpha_{s},\alpha_{\gamma }\right)=p\left(X|\bar{S},\beta ,\gamma ,\alpha_{0}\right)p\left(\bar{S}|\alpha_{s}\right)p\left(\gamma |\alpha_{\gamma }\right)p\left(\alpha_{0}\right)p\left(\alpha_{s}\right)p\left(\alpha_{\gamma }\right)p\left(\beta \right).$$

According to the received data X and the parameters of the prior distribution, the posterior distribution of $\bar{S}$ can be represented as

(29) $$\begin{array}{l} p\left(\bar{S}|X,\beta ,\gamma ,\alpha_{0},\alpha_{s},\alpha_{\gamma }\right)=\frac{p\left(X|\bar{S},\beta ,\gamma ,\alpha_{0},\alpha_{s},\alpha_{\gamma }\right)p\left(\bar{S}|\alpha_{s}\right)}{p\left(X|\beta ,\gamma ,\alpha_{0},\alpha_{s},\alpha_{\gamma }\right)}\\ \propto p\left(X|\bar{S},\beta ,\gamma ,\alpha_{0},\alpha_{s},\alpha_{\gamma }\right)p\left(\bar{S}|\alpha_{s}\right) \end{array},$$

where

(30) $$\begin{array}{cc} p\left(X|\bar{S},\beta ,\gamma ,\alpha_{0},\alpha_{s},\alpha_{\gamma }\right) & =\prod_{t=1}^{T}CN\left(y_{t}|\Psi \left(\beta \right)\left({\bar{s}}_{t}⊗\gamma \right),\alpha_{0}^{-1}I\right)\\ & =\prod_{t=1}^{T}\frac{\alpha_{0}^{M}}{\pi^{M}}\exp\left\{-\alpha_{0}{\left\|y_{t}-\Psi \left(\beta \right)\left({\bar{s}}_{t}⊗\gamma \right)\right\|}_{2}^{2}\right\} \end{array},$$

(31) $$\begin{array}{cc} p\left(\bar{S}|\alpha_{s}\right) & =\prod_{t=1}^{T}CN\left({\bar{s}}_{t}|0,\mathrm{diag}\left(\alpha_{s}\right)\right)\\ & =\prod_{t=1}^{T}\left(\prod_{n=1}^{N}\alpha_{sn}\right)\frac{1}{\pi^{N}}\exp\left\{-{\bar{s}}_{t}^{H}\mathrm{diag}\left(\alpha_{s}\right){\bar{s}}_{t}\right\} \end{array}.$$

Both $p\left(\bar{S}|\alpha_{s}\right)$ and $p\left(X|\bar{S},\beta ,\gamma ,\alpha_{0},\alpha_{s},\alpha_{\gamma }\right)$ are Gaussian functions; therefore, the posterior function of $p\left(\bar{S}|X,\beta ,\gamma ,\alpha_{0},\alpha_{s},\alpha_{\gamma }\right)$ can also be expressed as a Gaussian function:

(32) $$p\left(\bar{S}|X,\beta ,\gamma ,\alpha_{0},\alpha_{s},\alpha_{\gamma }\right)=\prod_{t=1}^{T}CN\left({\bar{s}}_{t}|\mu_{t},\Sigma_{s}\right),$$

where

(33) $$\mu_{t}=\alpha_{0}^{H}\Sigma_{s}^{H}\Omega^{H}\left(\beta ,\gamma \right)x_{t},$$

(34) $$\Sigma_{s}={\left[\alpha_{0}\Omega^{H}\left(\beta ,\gamma \right)\Omega \left(\beta ,\gamma \right)+\mathrm{diag}\left(\alpha_{s}\right)\right]}^{-1},$$

The next step is hyperparameter learning, which is equivalent to maximizing the posterior probability distribution of all the parameters to be estimated. In order to use Equation (28) to estimate other unknown parameters, the following likelihood can be used:

(35) $$\begin{array}{c} L\left(\beta ,\gamma ,\alpha_{0},\alpha_{s},\alpha_{\gamma }\right)\\ =\varepsilon \left\{\ln p\left(X|\bar{S},\beta ,\gamma ,\alpha_{0},\alpha_{s},\alpha_{\gamma }\right)p\left(\bar{S}|\alpha_{s}\right)p\left(\gamma |\alpha_{\gamma }\right)p\left(\alpha_{0}\right)p\left(\alpha_{s}\right)p\left(\alpha_{\gamma }\right)p\left(\beta \right)\right\} \end{array},$$

where ε{⋅} represents $\varepsilon_{\bar{S}|X,\beta ,\gamma ,\alpha_{n},\alpha_{s},\alpha_{\gamma }}\left\{\cdot \right\}$, which stands for the likelihood function under the expectation with respect to the posterior of $\bar{S}$.

(1) For the noise precision $\alpha_{0}$. Ignoring the unrelated terms, the likelihood function of $\alpha_{0}$ can be expressed as follows:

(36) $$\begin{array}{cc} L\left(\alpha_{0}\right) & =\varepsilon \left\{\ln p\left(X|\bar{S},\beta ,\gamma ,\alpha_{0}\right)p\left(\alpha_{0}\right)\right\}\\ & =\varepsilon \left\{\ln\prod_{t=1}^{T}CN\left(x_{t}|\Psi \left(\beta \right)\left({\bar{s}}_{t}⊗\gamma \right),\alpha_{0}^{-1}I\right)\right\}+\ln\Gamma \left(\alpha_{0};a,b\right) \end{array}.$$

Setting the derivative $\frac{\partial L\left(\alpha_{0}\right)}{\partial \alpha_{0}}$ to zero, solving for $\alpha_{0}$ with the given updates:

(37) $$\alpha_{0}=\frac{TM+a-1}{T\times Tr\left\{\Omega^{H}\left(\beta ,\gamma \right)\Omega \left(\beta ,\gamma \right)\Sigma_{s}\right\}+{\sum_{t=1}^{T}\left\|x_{t}-\Psi \left(\beta \right)\left(\mu_{t}⊗\gamma \right)\right\|}_{2}^{2}+b}.$$

(2) For the signal precision $\alpha_{s}$. Ignoring the unrelated terms, the likelihood function of $\alpha_{s}$ can be expressed as follows:

(38) $$\begin{array}{cc} L\left(\alpha_{s}\right) & =\varepsilon \left\{\ln p\left(\bar{S}|\alpha_{s}\right)p\left(\alpha_{s}\right)\right\}\\ & =\varepsilon \left\{\ln\prod_{t=1}^{T}CN\left({\bar{s}}_{t}|0,\Lambda_{s}\right)\right\}+\ln\prod_{n=1}^{N}\Gamma \left(\alpha_{sn};c,d\right) \end{array}.$$

Setting the derivative $\frac{\partial L\left(\alpha_{s}\right)}{\partial \alpha_{s}}$ to zero, the nth update value of $\alpha_{s}$ can be solved as

(39) $$\alpha_{sn}=\frac{T+c-1}{d+T\Sigma_{s,n,n}+{\sum_{t=1}^{T}\left|\mu_{n,t}\right|}^{2}},$$

where $\Sigma_{s,n,n}$ refers to the nth row and nth column elements of $\Sigma_{s}$, and $\mu_{n,t}$ refers to the nth value of $\mu_{t}$.

(3) For the gain-phase error vector γ. Ignoring the unrelated terms, the likelihood function of γ can be expressed as follows:

(40) $$\begin{array}{l} L\left(\gamma \right)=\varepsilon \left\{\ln p\left(X|\bar{S},\beta ,\gamma ,\alpha_{0}\right)p\left(\gamma |\alpha_{\gamma }\right)\right\}\\ =\varepsilon \left\{\ln\prod_{t=1}^{T}CN\left(x_{t}|\Psi \left(\beta \right)\left({\bar{s}}_{t}⊗\gamma \right),\alpha_{0}^{-1}I\right)\right\}+\ln\prod_{m=1}^{M}CN\left(\gamma_{m}|0,\alpha_{\gamma m}\right)\\ =TM\ln\frac{\alpha_{0}}{\pi }-\alpha_{0}T\cdot Tr\left\{\Omega^{H}\left(\beta ,\gamma \right)\Omega \left(\beta ,\gamma \right)\Sigma_{s}\right\}\\ -\alpha_{0}\sum_{t=1}^{T}{\left\|x_{t}-\Psi \left(\beta \right)\left(\mu_{t}⊗\gamma \right)\right\|}_{2}^{2}-\sum_{m=1}^{M}\alpha_{\gamma m}{\left|\gamma_{m}\right|}^{2}\\ \propto -\alpha_{0}T\cdot Tr\left\{\Omega^{H}\left(\beta ,\gamma \right)\Omega \left(\beta ,\gamma \right)\Sigma_{s}\right\}\\ -\alpha_{0}\sum_{t=1}^{T}{\left\|x_{t}-\Psi \left(\beta \right)\left(\mu_{t}⊗\gamma \right)\right\|}_{2}^{2}-\sum_{m=1}^{M}\alpha_{\gamma m}{\left|\gamma_{m}\right|}^{2} \end{array}.$$

To simplify this complicated equation, let us set

(41) $$Q_{1}\left(\beta ,\gamma \right)=Tr\left\{\Omega^{H}\left(\beta ,\gamma \right)\Omega \left(\beta ,\gamma \right)\Sigma_{s}\right\},$$

(42) $$Q_{2,t}\left(\beta ,\gamma \right)={\left\|x_{t}-\Psi \left(\beta \right)\left(\mu_{t}⊗\gamma \right)\right\|}_{2}^{2},$$

(43) $$Q_{3}\left(\gamma \right)=\sum_{m=1}^{M}\alpha_{\gamma m}{\left|\gamma_{m}\right|}^{2}$$

Calculating $\frac{\partial L\left(\gamma \right)}{\partial \gamma }$ according to the above equation, the following simplification can be obtained:

(44) $$\frac{\partial L\left(\gamma \right)}{\partial \gamma }=-\alpha_{0}T\frac{\partial Q_{1}\left(\beta ,\gamma \right)}{\partial \gamma }-\sum_{t=1}^{T}\alpha_{0}\frac{\partial Q_{2,t}\left(\beta ,\gamma \right)}{\partial \gamma }-\frac{\partial Q_{3}\left(\gamma \right)}{\partial \gamma }.$$

For Equation (44), we calculate $\frac{\partial Q_{1}\left(\beta ,\gamma \right)}{\partial \gamma }$, $\frac{\partial Q_{2,t}\left(\beta ,\gamma \right)}{\partial \gamma }$ and $\frac{\partial Q_{3}\left(\gamma \right)}{\partial \gamma }$ separately. For $\frac{\partial Q_{1}\left(\beta ,\gamma \right)}{\partial \gamma }$, first compute the mth value of $\frac{\partial Q_{1}\left(\beta ,\gamma \right)}{\partial \gamma }$:

(45) $$\begin{array}{l} {\left[\frac{\partial Q_{1}\left(\beta ,\gamma \right)}{\partial \gamma }\right]}_{m}=Tr\left\{\frac{\partial \Omega^{H}\left(\beta ,\gamma \right)\Omega \left(\beta ,\gamma \right)\Sigma_{s}}{\partial \gamma_{m}}\right\}\\ =Tr\left\{\frac{\partial {\left(I_{N}⊗\gamma \right)}^{H}}{\partial \gamma_{m}}\Psi^{H}\left(\beta \right)\Omega \left(\beta ,\gamma \right)\Sigma_{s}+\Omega^{H}\left(\beta ,\gamma \right)\Psi \left(\beta \right)\frac{\partial \left(I_{N}⊗\gamma \right)}{\partial \gamma_{m}}\Sigma_{s}\right\}\\ =Tr\left\{\Omega^{H}\left(\beta ,\gamma \right)\Omega \left(\beta ,e_{m}^{M}\right)\Sigma_{s}\right\}\\ =\gamma^{H}\left(\sum_{p=1}^{N}\sum_{k=1}^{N}\Psi_{p}^{H}\left(\beta \right)\Psi_{k}\left(\beta \right)\Sigma_{s,k,p}\right)e_{m}^{M} \end{array},$$

where $e_{m}^{M}$ is the M×1 dimensional vector with the mth entry being 1 and the other entries being 0. Therefore, we can obtain

(46) $$\frac{\partial Q_{1}\left(\beta ,\gamma \right)}{\partial \gamma }=\gamma^{H}\left(\sum_{p=1}^{N}\sum_{k=1}^{N}\Psi_{p}^{H}\left(\beta \right)\Psi_{k}\left(\beta \right)\Sigma_{s,k,p}\right).$$

For $\frac{\partial Q_{2,t}\left(\beta ,\gamma \right)}{\partial \gamma }$, we can simplify it to

(47) $$\begin{array}{cc} \frac{\partial Q_{2,t}\left(\beta ,\gamma \right)}{\partial \gamma } & =-{\left[x_{t}-\Psi \left(\beta \right)\left(\mu_{t}⊗\gamma \right)\right]}^{H}\Psi \left(\beta \right)\frac{\partial \mu_{t}⊗\gamma }{\partial \gamma }\\ & =-{\left[x_{t}-\Psi \left(\beta \right)\left(\mu_{t}⊗\gamma \right)\right]}^{H}\Psi \left(\beta \right)\left(\mu_{t}⊗I_{M}\right) \end{array}.$$

For $\frac{\partial Q_{3}\left(\gamma \right)}{\partial \gamma }$, we can simplify it to

(48) $$\frac{\partial Q_{3}\left(\gamma \right)}{\partial \gamma }=\gamma^{H}\mathrm{diag}\left(\alpha_{\gamma }\right).$$

By setting $\frac{\partial L\left(\gamma \right)}{\partial \gamma }=0$, we can obtain

(49) $$\gamma =H^{-1}d,$$

where

(50) $$H=\sum_{t=1}^{T}\alpha_{0}\Xi^{H}\left(\beta ,\mu_{t}\right)\Xi \left(\beta ,\mu_{t}\right)+\alpha_{0}T{\left(\sum_{p=0}^{N}\sum_{k=0}^{N}\Psi_{p}^{H}\left(\beta \right)\Psi_{k}\left(\beta \right)\Sigma_{s,k,p}\right)}^{H}+\mathrm{diag}\left(\alpha_{\gamma }\right),$$

(51) $$d=\sum_{t=1}^{T}\alpha_{0}\Xi^{H}\left(\beta ,\mu_{t}\right)x_{t},$$

where $\Xi \left(\beta ,\mu_{t}\right)≜\Psi \left(\beta \right)\left(\mu_{t}⊗I_{M}\right)$.

(4) For the gain-phase error precision $\alpha_{\gamma }$. Ignoring the independent terms, we can obtain the following likelihood function of $\alpha_{\gamma }$:

(52) $$\begin{array}{cc} L\left(\alpha_{\gamma }\right) & =\varepsilon \left\{\ln p\left(\gamma |\alpha_{\gamma }\right)p\left(\alpha_{\gamma }\right)\right\}\\ & =\varepsilon \left\{\ln\prod_{m=0}^{M}CN\left(\gamma_{m}|0,\alpha_{\gamma m}\right)\right\}+\ln\prod_{m=0}^{M}\Gamma \left(\alpha_{\gamma m};e,f\right) \end{array}.$$

Let $\frac{\partial L\left(\alpha_{\gamma }\right)}{\partial \alpha_{\gamma }}=0$; then, we can obtain a newer version of the mth value of $\alpha_{\gamma }$:

(53) $$\alpha_{\gamma m}\approx \frac{1}{f+\gamma_{n}^{H}\gamma_{n}}.$$

(5) For the off-grid vector β. Ignoring the independent terms, we can obtain the following likelihood function of β:

(54) $$\begin{array}{l} L\left(\beta \right)=\varepsilon \left\{\ln p\left(X|\bar{S},\beta ,\gamma ,\alpha_{0}\right)p\left(\beta \right)\right\}\\ \begin{array}{cc} & \begin{array}{cc} & \propto -TQ_{1}\left(\beta ,\gamma \right) \end{array} \end{array}-\sum_{t=1}^{T}Q_{2,t}\left(\beta ,\gamma \right) \end{array}.$$

Calculate $\frac{\partial L\left(\beta \right)}{\partial \beta }$ according to the above equation, and similarly calculate $\frac{\partial Q_{1}\left(\beta ,\gamma \right)}{\partial \beta }$ and $\frac{\partial Q_{2,t}\left(\beta ,\gamma \right)}{\partial \beta }$. First, compute the nth value of $\frac{\partial Q_{1}\left(\beta ,\gamma \right)}{\partial \beta }$:

(55) $$\begin{array}{cc} {\left[\frac{\partial Q_{1}\left(\beta ,\gamma \right)}{\partial \beta }\right]}_{n} & =Tr\left\{\frac{\partial \Omega^{H}\left(\beta ,\gamma \right)\Omega \left(\beta ,\gamma \right)\Sigma_{s}}{\partial \beta_{m}}\right\}\\ & =Tr\left\{\left[0,\Omega^{H}\left(\beta ,\gamma \right)B_{\Lambda n}\gamma ,0\right]\Sigma_{s}\right\}+Tr\left\{{\left[0,\Omega^{H}\left(\beta ,\gamma \right)B_{\Lambda n}\gamma ,0\right]}^{H}\Sigma_{s}\right\}\\ & =2ℜ\left\{{\left[\Omega^{H}\left(\beta ,\gamma \right)B_{\Lambda n}\gamma \right]}^{H}\Sigma_{s,:,n}\right\} \end{array}.$$

Then, $\frac{\partial Q_{1}\left(\beta ,\gamma \right)}{\partial \beta }$ can be simplified as

(56) $$\frac{\partial Q_{1}\left(\beta ,\gamma \right)}{\partial \beta }=2ℜ\left\{\mathrm{diag}\left(\Sigma_{s}\Omega^{H}\left(\beta ,\gamma \right)B_{\Lambda }\left(I_{N}⊗\gamma \right)\right)\right\}.$$

For $\frac{\partial Q_{2,t}\left(\beta ,\gamma \right)}{\partial \beta }$, we can simplify it to

(57) $$\begin{array}{cc} \frac{\partial Q_{2,t}\left(\beta ,\gamma \right)}{\partial \beta } & =-2ℜ\left\{{\left[x_{t}-\Psi \left(\beta \right)\left(\mu_{t}⊗\gamma \right)\right]}^{H}\frac{\partial \Psi \left(\beta \right)\left(\mu_{t}⊗\gamma \right)}{\partial \beta }\right\}\\ & =-2ℜ\left\{{\left[x_{t}-\Psi \left(\beta \right)\left(\mu_{t}⊗\gamma \right)\right]}^{H}B_{\Lambda }\left(\mathrm{diag}\left(\mu_{t}\right)⊗\gamma \right)\right\} \end{array}.$$

By setting $\frac{\partial L\left(\beta \right)}{\partial \beta }=0$, we can obtain

(58) $$\beta =P^{-1}v,$$

where

(59) $$P_{n,:}=ℜ\left\{T\gamma^{H}B_{\Lambda n}^{H}B_{\Lambda }\left(\mathrm{diag}\left(\Sigma_{s,:,n}\right)⊗\gamma \right)\right\}+\sum_{t=1}^{T}ℜ\left\{\mu_{t,n}\gamma^{T}B_{\Lambda n}^{H}B_{\Lambda }^{*}\left(I_{N}⊗\gamma^{*}\right){\mathrm{diag}}^{*}\left(\mu_{t}\right)\right\},$$

(60) $$v_{n}=\sum_{t=1}^{T}ℜ\left\{{\left[x_{t}-A_{\Lambda }\left(\mu_{t}⊗\gamma \right)\right]}^{H}B_{\Lambda n}\mu_{t,n}\gamma \right\}-Tℜ\left\{\gamma^{H}B_{\Lambda n}^{H}A_{\Lambda }\left(I_{N}⊗\gamma \right)\Sigma_{s,:,n}\right\}.$$

## 4. Matching of Elevation Angle and Azimuth Angle

In most existing compressed sensing DOA estimation methods based on L-shaped arrays, one-dimensional DOA is used to estimate the elevation angle and azimuth angles, and then the elevation and azimuth angles are matched. The process for this kind of method is complicated and the estimation accuracy is not high. Therefore, in order to solve this problem, an auxiliary angle is proposed to realize the automatic matching of elevation and azimuth angles.

According to Equations (7) and (8), the cross-correlation covariance matrix of the two subarrays can be expressed as

(61) $$R_{zy}=Ε\left[z\left(t\right)y^{H}\left(t\right)\right]=\;\Gamma \;\prime \;A\left(\theta \right)R_{s}A^{H}\left(\phi \right),$$

where $R_{s}=Ε\left[s\left(t\right)s^{H}\left(t\right)\right]$; we then take the diagonal elements of its cross-correlation covariance matrix:

(62) $$\begin{array}{cc} r_{zy} & =\mathrm{diag}\left(R_{zy}\right)\\ & =\;\Gamma \;\prime \left[\sum_{k=1}^{K}\delta_{k},\sum_{k=1}^{K}\delta_{k}e^{\frac{-j2\pi d\left(\cos\phi_{k}-\cos\theta_{k}\right)}{\lambda }},\cdots ,\sum_{k=1}^{K}\delta_{k}e^{\frac{-j2\pi d\left(M-1\right)\left(\cos\phi_{k}-\cos\theta_{k}\right)}{\lambda }}\right]\\ & =\;\Gamma \;\prime \left[\sum_{k=1}^{K}\delta_{k},\sum_{k=1}^{K}\delta_{k}e^{\frac{-j2\pi d\cos\eta_{k}}{\lambda }},\cdots ,\sum_{k=1}^{K}\delta_{k}e^{\frac{-j2\pi d\left(M-1\right)\cos\eta_{k}}{\lambda }}\right]\\ & =\;\Gamma \;\prime \;A\left(\eta \right)\delta \end{array},$$

where $\;\Gamma \;\prime =\Gamma_{z}\Gamma_{y}$, $\delta =\left[\delta_{1},\delta_{2},\cdots ,\delta_{K}\right]$ and $\delta_{k}$ represents the power of the kth incident signal $s_{k}\left(t\right)$, which is the kth diagonal element of $R_{s}$. Equation (62) can be regarded as the received signal with angle parameter $\eta_{k}$ incident on a one-dimensional array of M sensor elements, which satisfies the array signal model of Equation (10). Therefore, the auxiliary angle $\eta_{k}$ can be solved using the one-dimensional sparse Bayesian self-calibration DOA estimation method described in Section 2.

Then, according to Equation (9), the $\eta_{k}$ obtained by the solution is substituted into the expression of the signal received by the array:

(63) $$\begin{array}{cc} X & =\Gamma A\left(\theta ,\phi \right)S+N=\Gamma \Theta \left(\eta \right)\left(I_{K}⊗A\left(\theta \right)\right)S+N\\ & =\Gamma \tilde{A}\left(\theta \right)S+N \end{array},$$

where $\Theta \left(\eta \right)=\left[\Theta \left({\hat{\eta }}_{1}\right),\Theta \left({\hat{\eta }}_{2}\right),\cdots ,\Theta \left({\hat{\eta }}_{K}\right)\right]$ and ${\hat{\eta }}_{k}$ represents the estimated value of the solved auxiliary angle $\eta_{k}$.

Equation (63) also satisfies the array signal model of Equation (10), and can also be solved using the one-dimensional sparse Bayes self-correcting DOA estimation method in Section 2; then, the corresponding block sparse estimation results are obtained. For each “block”, the sparse signal value is recovered by searching the spectral peak, and the elevation angle $\theta_{k}$ corresponding to the auxiliary angle $\eta_{k}$ is estimated. Finally, according to the relationship between the three angles, the corresponding azimuth angle estimate can be obtained as

(64) $${\hat{\phi }}_{k}=\mathrm{argcos}\left(\cos{\hat{\theta }}_{k}+{\hat{\eta }}_{k}\right).$$

## 5. Simulation Results

Simulation experiments were conducted to compare the proposed method with the 2D-MUSIC method, OMP method and OGSBI method, so as to verify the estimation performance of the proposed method. With reference to the relevant literature, the gain-phase error of the array was assumed to conform to the following model:

(65) $$\{\begin{array}{c} \rho_{m}=1+\sqrt{12}\sigma_{\rho }\kappa_{m}\\ \varphi_{m}=\sqrt{12}\sigma_{\varphi }\zeta_{m} \end{array},$$

where $\sigma_{\rho }$ and $\sigma_{\varphi }$ stand for the standard deviations of gain errors and phase errors, respectively, and $\kappa_{m}$ and $\zeta_{m}$ are independent random variables distributed uniformly over [−0.5,0.5].

**Figure 2:**
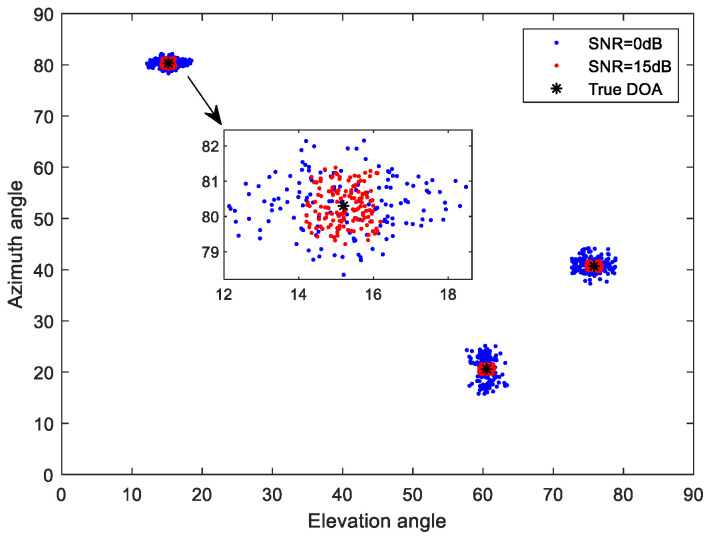
Spatial estimation of the proposed method.

**Figure 3:**
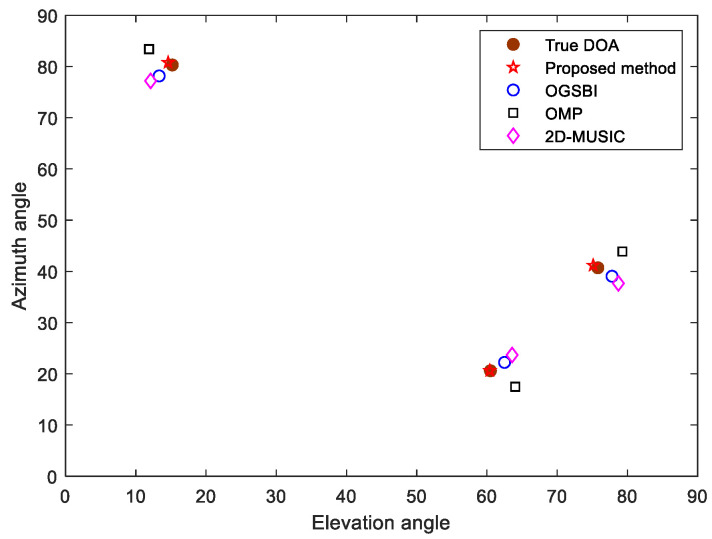
Spatial estimation using different methods.

Consider a uniform L-shaped array with M=8 array elements on the subarray, with an array element spacing of half the wavelength; the wavelength was set to 0.06, and the three DOAs of the far-field narrow band signal impacting the array were $\left(\theta_{1},\phi_{1}\right)=\left(15.2{}^{\circ},80.3{}^{\circ}\right)$, $\left(\theta_{2},\phi_{2}\right)=\left(60.5{}^{\circ},20.6{}^{\circ}\right)$ and $\left(\theta_{3},\phi_{3}\right)=\left(75.8{}^{\circ},40.7{}^{\circ}\right)$. $\sigma_{\rho }=\sigma_{\varphi }=0.5$, the number of snapshots was 100, and the grid interval was 1°. The noise was additive white Gaussian noise. The estimation results of 150 Monte Carlo experiments under the condition of SNR = 0 dB and SNR = 15 dB are shown in Figure 2. Figure 3 shows the spatial estimation results obtained by the proposed method, 2D-MUSIC method, OMP method and OGSBI method when the SNR was 10 dB.

As can be seen from Figure 2, when the SNR was 15 dB, the DOA estimation result of the proposed method was close to the true direction of the incident source. When the SNR was 0 dB, although the estimation accuracy was reduced, the approximate location of the source was still effectively estimated. Compared with the other three methods, the proposed method reduced the influence of the gain-phase errors, and its estimate was the closest to the true source position (Figure 3).

The root mean square error (RMSE) was used to evaluate the estimation errors of the proposed method and the 2D-MUSIC, OMP and OGSBI methods. The number of Monte Carlo simulation experiments was set to I=300, and the root-mean-square error of the elevation angle and the azimuth angle estimations of K incident signals are defined as

(66) $$RMSE1=\sqrt{\frac{1}{2IK}\sum_{i=1}^{I}\sum_{k=1}^{K}{\left({\hat{\theta }}_{k}^{i}-\theta_{k}\right)}^{2}}$$

(67) $$RMSE2=\sqrt{\frac{1}{2IK}\sum_{i=1}^{I}\sum_{k=1}^{K}{\left({\hat{\phi }}_{k}^{i}-\phi_{k}\right)}^{2}}$$

where ${\hat{\theta }}_{k}^{i}$ represents the estimated azimuth angle of the kth signal in the ith Monte Carlo simulation experiment and ${\hat{\phi }}_{k}^{i}$ represents the estimated elevation angle of the kth signal in the ith Monte Carlo simulation experiment.

**Figure 4:**
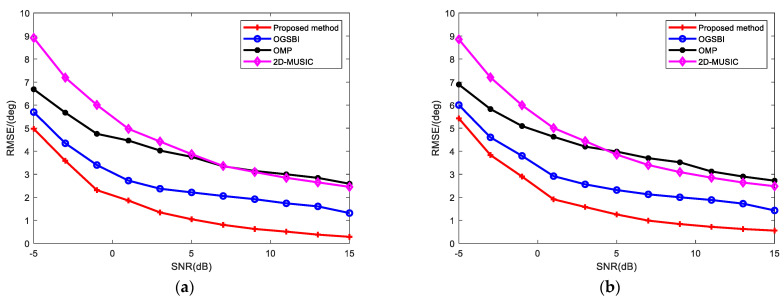
RMSE vs. SNR for (**a**) elevation angle; (**b**) azimuth angle.

Figure 4 shows the RMSEs of the OGSBI method, OMP method, 2D-MUSIC method and the proposed method when the SNR was changed without affecting any of the other settings. It can be seen that the RMSE curves of the four methods all decreased gradually with increasing SNR. The RMSE curves of the 2D-MUSIC and OMP methods were higher and the error was large. The proposed method was superior to the other methods in this SNR interval with the lowest RMSE curve and the smallest error. When the SNR was 15 dB, not only the RMSE of the elevation angle, but also the RMSE of the azimuth angle were at the lowest values, only 0.2838 and 0.5537. This shows that when the SNR is high, the advantages of the proposed method are more obvious.

**Figure 5:**
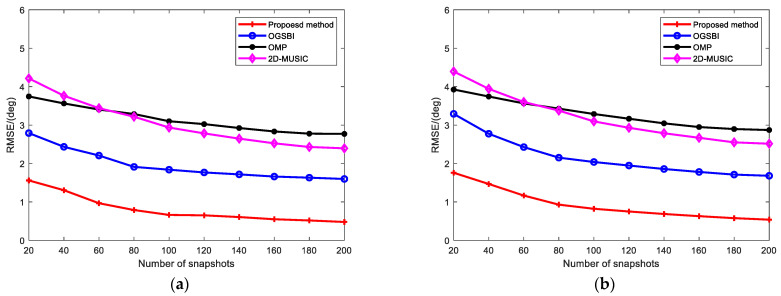
RMSE vs. number of snapshots for (**a**) elevation angle; (**b**) azimuth angle.

Figure 5 shows the RMSEs of the four methods when the SNR was set to 10 dB and the other experimental conditions were unchanged. The RMSEs of the four methods showed a decreasing trend with increasing number of snapshots. The RMSE curve of the proposed method was lower than that of the other three methods, and the RMSEs of the elevation and azimuth angles were only 1.5596 and 1.7965 when the number of snapshots was 20. This shows that the proposed method has the best estimation performance under different snapshot numbers compared to the other methods.

**Figure 6:**
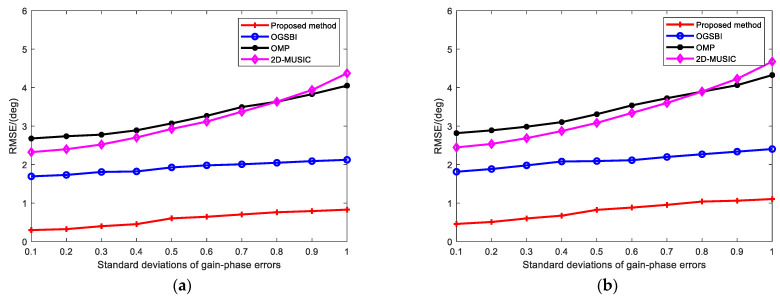
RMSE vs. standard deviation of gain-phase errors for (**a**) elevation angle; (**b**) azimuth angle.

Figure 6 shows the RMSE of the four methods when, with the other experimental conditions unchanged, the SNR was set to 10 dB, the number of snapshots was 100, and the magnitude of the gain-phase error (i.e., the standard deviation coefficient of the gain-phase error) was changed. The RMSEs of the four methods increased with the increase in gain-phase errors, but the proposed method was less affected by the magnitude of the gain-phase errors, and the RMSE curve remained the lowest. The 2D-MUSIC method was significantly affected by gain-phase errors, and its RMSE fluctuated the most. This shows that, compared to other methods, the proposed method can effectively reduce the influence of gain-phase errors and has the best estimation performance.

The angular resolution ability is another important index that can be used to judge the performance of DOA estimation, and the resolution ability of different methods is different. In this study, the angular resolution ability of the proposed method was evaluated from the perspective of SNR and the standard deviation coefficient of the gain-phase error. The method can identify the source position correctly, when the following conditions are met:

(68) $$\max\left\{\left|{\bar{\theta }}_{k}-\theta_{k}\right|,\left|{\bar{\phi }}_{k}-\phi_{k}\right|\right\}<\varepsilon_{e},$$

where ${\bar{\theta }}_{k}$ is the estimate of the true azimuth angle $\theta_{k}$, ${\bar{\phi }}_{k}$ is the estimate of the true elevation angle $\phi_{k}$, and $\varepsilon_{e}$ represents the set angle error threshold, which is set to 1 here.

**Figure 7:**
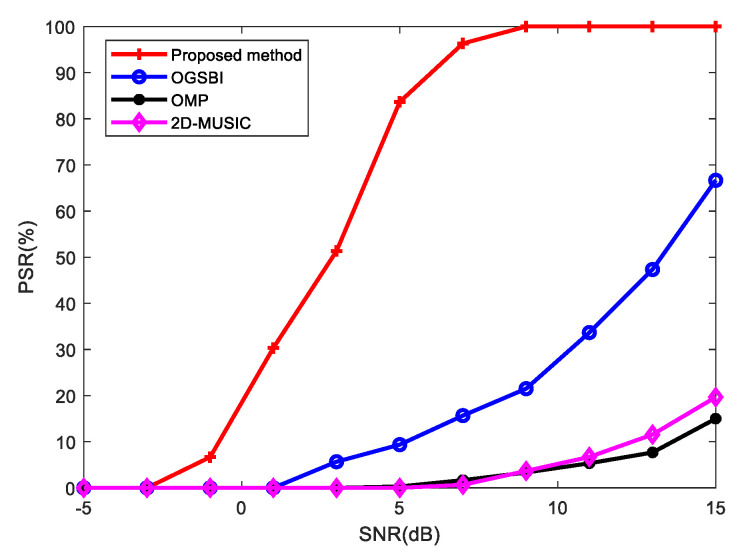
PSR vs. SNR.

Keeping the other experimental conditions unchanged, the number of snapshots was set to 100, $\sigma_{\rho }=\sigma_{\varphi }=0.5$, and the SNR was varied. The results of the probability of successful resolution (PSR) of the OGSBI method, OMP method, 2D-MUSIC method and the proposed method are shown in Figure 7.

It can be seen from Figure 7 that the PSR of the proposed method was higher than that of the other methods in both low and high SNR scenarios. The PSR of the proposed method reached 100% when the SNR was 9 dB, while the other three methods did not reached 100% even when the SNR was 15 dB, and their success probability of resolution was low, and their resolution ability was poor. This shows that the proposed method has better angular resolution and robustness compared to the other methods under different SNR conditions.

**Figure 8:**
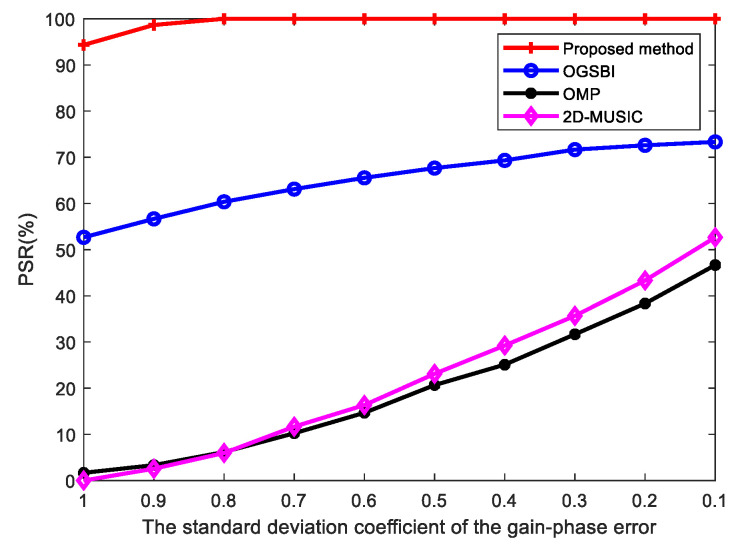
PSR vs. standard deviation of gain-phase error.

In Figure 8, the PSRs of the four methods are compared when, keeping the other experimental conditions unchanged, the number of snapshots was set to 100, the SNR was set to 15 dB, and the standard deviation coefficients of the gain-phase error $\sigma_{\rho }$ and $\sigma_{\rho }$ were changed. The figure shows that, in the case of large gain-phase errors, the PSRs of the other methods were low, and the OMP and 2D-MUSIC methods could not successfully predict the angles, while the PSR of the proposed method reached 66.7%. In the case of small gain-phase errors, the PSR of the proposed method reached 100%, which is obviously better than the other methods.

## 6. Conclusions

In order to solve the problems of low accuracy and poor angular resolution caused by unknown gain-phase errors, a robust sparse Bayesian two-dimensional DOA estimation method for scenarios with gain-phase errors was proposed in this paper. To avoid the additional angle matching problem in the traditional method, the proposed method introduces an auxiliary angle to match the elevation angle and the azimuth angle. A new sparse representation model with gain-phase errors is constructed by using the diagonal elements of the cross-correlation matrix received by two submatrices, and auxiliary angles are obtained by sparse Bayes inference. By substituting in the obtained auxiliary angles, a new sparse representation model is constructed again according to the relationship between the three angles, and the corresponding block sparse estimation results are obtained. For each “block”, the corresponding DOA is obtained by searching the spectral peak. The simulation results show that the DOA estimation accuracy and angle resolution of the proposed method are superior to the OGSBL, OMP and 2D-MUSIC methods in cases where gain-phase errors exist.

## Figures and Tables

**Table 1:** Mathematical symbols and their meanings.

Symbol	Representative Meaning
${\left(\cdot \right)}^{T}$	Transpose
${\left(\cdot \right)}^{H}$	Conjugate transpose
${\left(\cdot \right)}^{*}$	Conjugate
${\left(\cdot \right)}^{-1}$	Matrix inversion
diag(⋅)	Diagonal matrix
blkdiag(⋅)	Block diagonal matrix
ℜ{⋅}	Real part of the complex value
Tr{⋅}	Trace operation of a matrix
${\left\|\cdot \right\|}_{2}$	$l_{2}$ norm

## Data Availability

Data are contained within the article.
